# Zeolite Composite Materials from Fly Ash: An Assessment of Physicochemical and Adsorption Properties

**DOI:** 10.3390/ma16062142

**Published:** 2023-03-07

**Authors:** Jakub Mokrzycki, Wojciech Franus, Rafał Panek, Maciej Sobczyk, Piotr Rusiniak, Justyna Szerement, Renata Jarosz, Lidia Marcińska-Mazur, Tomasz Bajda, Monika Mierzwa-Hersztek

**Affiliations:** 1 Department of Coal Chemistry and Environmental Sciences, Faculty of Energy and Fuels, AGH University of Science and Technology, Mickiewicza 30 Av., 30-059 Cracow, Poland; 2 Department of Construction Materials Engineering and Geoengineering, Civil Engineering and Architecture Faculty, Lublin University of Technology, Nadbystrzycka 40, 20-618 Lublin, Poland; 3 Department of Mineralogy, Petrography and Geochemistry, Faculty of Geology, Geophysics and Environmental Protection, AGH University of Science and Technology, Mickiewicza 30 Av., 30-059 Cracow, Poland; 4 Department of Hydrogeology and Engineering Geology, Faculty of Geology, Geophysics and Environmental Protection, AGH University of Science and Technology, Mickiewicza 30 Av., 30-059 Cracow, Poland; 5 Department of Radiochemistry and Environmental Chemistry, Maria Curie–Skłodowska University, 3 Maria Curie–Skłodowska Square, 20-031 Lublin, Poland; 6 Department of Agricultural and Environmental Chemistry, University of Agriculture in Krakow, Mickiewicza 21 Av., 31-120 Cracow, Poland

**Keywords:** zeolite composites, fly ash, hydrothermal synthesis, ammonium ions adsorption

## Abstract

Waste fly ash, with both low (with the addition of vermiculite) and high contents of unburned coal, were subjected to hydrothermal syntheses aiming to obtain zeolite composite materials—zeolite + vermiculite (NaX–Ver) and zeolite + unburned carbon (NaX–C). The composites were compared with parent zeolite obtained from waste fly ash with a low content of unburned carbon (NaX–FA). In this study, the physicochemical characteristics of the obtained materials were evaluated. The potential application of the investigated zeolites for the adsorption of ammonium ions from aqueous solutions was determined. Composite NaX–Ver and parent zeolite NaX–FA were characterized by comparable adsorption capacities toward ammonium ions of 38.46 and 40.00 mg (NH_4_^+^) g^−1^, respectively. The nearly 2-fold lower adsorption capacity of composite NaX–C (21.05 mg (NH_4_^+^) g^−1^) was probably a result of the lower availability of ion exchange sites within the material. Adsorbents were also regenerated using 1 M NaCl solution at a pH of 10 and subjected to 3 cycles of adsorption–desorption experiments, which proved only a small reduction in adsorption properties. This study follows the current trend of waste utilization (fly ash) and the removal of pollutants from aqueous solutions with respect to their reuse, which remains in line with the goals of the circular economy.

## 1. Introduction

The global demand for energy production is constantly growing annually. However, despite strict regulations in terms of reducing carbon footprint and the global goal to become carbon-neutral, coal and oil remain the main sources of energy worldwide (57.7% in 2019) [1]. The production of energy from coal generates significant amounts of waste that can cause a threat to the environment. Despite carbon dioxide (CO_2_), the emissions are strictly regulated, and the by-product produced during combustion is solid waste known as coal fly ash (CFA) [2]. Its annual production worldwide is up to 800 Mt. According to the ASTM (American Society for Testing and Materials) classification, there are two classes of CFA, namely class C and class F [3]. CFA class C is formed after the burning of lignite and the sum of the most dominant oxides, namely SiO_2_, Al_2_O_3_, and Fe_2_O_3_ is at least 50%. The presence of CaO above 10% is responsible for the self-hardening of CFA class C. On the other hand, CFA class F is formed after the combustion of coal and the total content of SiO_2_, Al_2_O_3_, and Fe_2_O_3_ is at least 70%. CFA class F is also characterized by a significantly lower amount of CaO compared to CFA class C [4]. There have been numerous attempts to find practical uses for CFA, as it has become a problematic landfilled waste. In fact, nowadays, only about 25% of CFA is being utilized. The pathways for CFA utilization involve (i) the production of construction materials [5], (ii) the production of ceramics [6], (iii) the recovery of rare earth elements (REEs) [7], and (iv) soil amendment [8]. A promising approach for CFA utilization is its application as a feedstock for the production of value-added materials, namely zeolites [9], mesoporous silica [10], also metal–organic frameworks (MOFs) [11]. The technology is being constantly developed, despite it being known for nearly 25 years [12,13,14]. Zeolites are a group of aluminosilicate materials of a three-dimensional framework built from [SiO_4_]^4−^ and [AlO_4_]^5−^ tetrahedra units [15]. These tetrahedra units are combined by oxygen groups forming so-called cuboctahedrons of unique crystalline structures. In fact, there are about 247 different zeolite framework types. The negative charge located at [AlO_4_]^5−^ tetrahedra is compensated by alkali (Li^+^, Na^+^, K^+^) or alkali earth (Mg^2+^, Ca^2+^, Ba^2+^) metal ions. Due to their properties, including a uniform porous structure that forms cavities, channels, and tunnels of nanometric size, thermal and hydrothermal stability, and easy ion exchange, zeolites have found numerous applications [16,17]. The application of zeolites as catalyst supports [18], in the adsorption processes of various pollutants, both organic [19,20] and inorganic [21], and soil amendment [22] are the most common pathways of their use. In the papers of Fedyna et al. [18,23], the researchers investigated BEA–zeolite composite materials with AlSBA-15 to improve the share of multibranched or monobranched alkanes in the hydroisomerization of n-heptane or n-hexadecane, respectively. Wołowiec et al. [24] investigated the removal of volatile organic compounds (VOCs) as well as polycyclic aromatic hydrocarbons (PAHs) using surfactant-modified zeolites. It was proven that the modification of zeolites with hexadecyltrimethylammonium bromide (HDTMA) improved the removal of VOCs, whereas it had a rather negligible effect on the removal of PAHs. In our recent papers [21,25], we have investigated the removal of phosphate ions from an aqueous solution using ion-exchanged zeolites from fly ashes. It was proven that copper-ion-exchanged zeolite X, using a 0.05 M solution of copper(II) nitrate and a proton form of zeolite X, displayed improved adsorption capacities toward phosphate ions in comparison with parent zeolite NaX–FA by 20-fold and 8-fold, respectively. The application of zeolites in mineral–organic mixtures as fertilizers was also widely investigated, both before and after in vivo experiments with plants, to investigate their effect on soil microbial composition [26] and their effect on both plant growth and the translocation of heavy metals from soil to plant tissues [27].

It can be clearly concluded that to accelerate the properties of zeolites toward various applications, some modifications applied to their structure are required. A promising pathway is the preparation of composite materials, which can show a synergic effect of the phases [28]. Şimşek et al. [29] found that the addition of vermiculite—a phyllosilicate [30] clay mineral—to the polymer (polyacrylamide) positively affected its adsorption efficiency toward uranyl ions from aqueous solutions. However, to make sure that composite materials are safe, a screening investigation of their properties is mandatory prior to their application.

Ammonium ions (NH_4_^+^), which are extensively released into the environment, can cause a serious problem of eutrophication. The adsorption properties of zeolites toward ammonium ions from aqueous solutions are constantly being investigated. The performance of particular zeolites in ammonium ion removal depends on structure, chemical composition, and the treatment of the adsorbent [31]. Zeolite NaA has been reported to have an adsorption capacity of 37.81 mg (NH_4_^+^) g^−1^ [32]. However, the synthetic zeolite P was characterized by an adsorption capacity toward ammonium ions of only 13.63 mg (NH_4_^+^) g^−1^ [33]. The adsorption of ammonium ions by zeolites was also reported to depend upon the form of zeolite. Zeolite Y in sodium form displayed greater adsorption capacity toward ammonium ions when compared with Cs, Mg, Ca, and K forms [34].

This study aimed to synthesize zeolite materials using fly ashes. First, the zeolite NaX–FA (fly ash zeolite NaX) was synthesized as a reference material. Next, we investigated the composite materials (i) zeolite NaX–C (zeolite NaX composite with carbon) obtained from high carbon fly ash (HCFA), and (ii) NaX–Ver (zeolite NaX composite with vermiculite). The obtained materials were then characterized in detail by using XRD, XRF, FTIR, N_2_ adsorption–desorption, and SEM to obtain a better understanding of their chemical and physical properties. Aiming to evaluate the safety of the materials, the leaching test was performed along with the determination of PAHs (polycyclic aromatic hydrocarbons) and uranium radionuclide content. To the best of the authors’ knowledge, such detailed characteristics (in terms of PAHs and radionuclide content) of zeolites from fly ashes have not been reported in the literature. To evaluate the potential utility of zeolites, the materials were subjected to a series of adsorption experiments aiming to examine the ion exchange properties toward the removal of ammonium ions from aqueous solutions. Adsorption parameters, i.e., adsorbent dose and solution pH were also defined. The pseudo-first-order and pseudo-second-order adsorption kinetic models were employed to find the adsorption parameters. Adsorption statics were performed to find the maximum adsorption capacities of the investigated materials and the results were correlated with the adsorption isotherms of Langmuir and Freundlich. Moreover, the adsorbents were regenerated and subjected to three cycles of adsorption–desorption studies, implying highly efficient regeneration.

## 2. Materials and Methods

### 2.1. Materials Synthesis

The synthesis of zeolite NaX–FA was conducted using a hydrothermal method and a conventional coal fly ash (CFA) (Jaworzno Power Station, Jaworzno, Poland), derived from hard coal combustion (class F according to ASTM C 618-08), and NaOH solution (P.P.H. “Stanlab”, Lublin, Poland) as reagents. A technological line for zeolite synthesis was loaded with 25 kg of CFA and 90 dm^3^ of 3 M NaOH [14]. The reaction temperature of 80 °C was maintained for 48 h using three heaters of 3 kW. The mixing procedure was as follows: 5 min mixing every 30 min coupled with rotating for 10 min every 30 min. After the synthesis, the solid product was rinsed with distilled water and dried at 105 °C.

The zeolite composite (NaX–Ver) synthesis was carried out using the liquid waste obtained during the synthesis of the NaX–FA zeolite described above. This waste is a solution rich in silicon and aluminum resulting from the dissolution of an aluminosilicate glaze contained in fly ash in a concentrated NaOH(aq) solution. Hence, it allows reusing spent waste solution as a feedstock for a new synthesis. First, 45 dm^3^ of the waste solution from the zeolite NaX–FA synthesis was transferred into the reactor (technological line), with the addition of 1.2 kg of vermiculite (Namekara mine, Mbale, Uganda) (fractions medium:fine:superfine ratio was 1:2:1) [14]. The reagents were mixed for 24 h (5 min in every 2 h). This operation was used due to the expanded nature of the vermiculite structure, which allowed the waste solution to be introduced between successive layers of the mineral. Next, 45 dm^3^ of 2 M NaOH solution (P.P.H. “Stanlab”, Lublin, Poland) was added to the reactor. Then, the hydrothermal synthesis was conducted for 24 h at 70 °C. The reaction temperature was maintained by three heaters of 3 kW total. The reaction mixture was mixed as follows: 30 s every 15 min coupled with circulation for 1 min every 30 min. After the synthesis, the solid product was filtered in a hydraulic press. Then, the product was rinsed with water (tap and distilled) and dried at 105 °C.

The synthesis of the zeolite–carbon composite (NaX–C) was conducted using a hydrothermal method and a high carbon fly ash (HCFA) from hard coal combustion (class F according to ASTM C 618-08) collected from a thermal power plant (Janikowo, Poland), after the electromagnetic separation of the fly ash, and NaOH solution (P.P.H. “Stanlab”, Lublin, Poland) as reagents [35]. A technological line for zeolite synthesis was loaded with 20 kg of HCFA and 90 dm^3^ of 3 M NaOH [14]. The reaction temperature of 70 °C was maintained for 48 h using three heaters of 3 kW. The mixing procedure was as follows: 5 min of mixing in a 1 h interval coupled with rotating for 10 min every hour. After the synthesis, the solid product was rinsed with distilled water and dried at 105 °C.

**Scheme 1:**
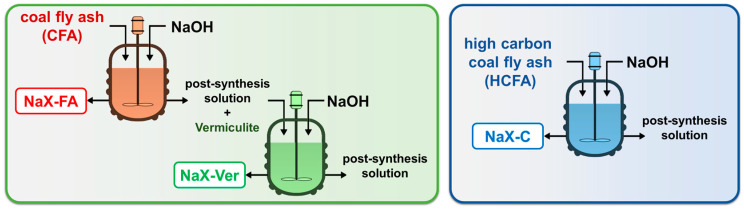
The schematic diagram for zeolite NaX–FA and zeolite composites NaX–Ver and NaX–C syntheses.

The chemical compositions of CFA, HCFA, and vermiculite are summarized in Table S1. The schematic illustration of the zeolite syntheses is presented in Scheme 1.

### 2.2. Materials Characterization

#### 2.2.1. X-ray Diffraction (XRD)

Powder X-ray diffraction (XRD) patterns were obtained using a Rigaku SmartLab diffractometer (Neu-Isenburg, Tokyo, Japan). The 2θ range varied from 5 to 75° with a constant step of 0.02° using graphite–monochromatized CuKα radiation. Prior to the analyses, the samples were ground using a mortar. Phase identification was performed using XRAYAN software (v 4.0.5, “KOMA”—Henryk Marciniak, Warszawa, Poland) based on the International Centre for Diffraction Data (ICDD) powder diffraction pattern database.

#### 2.2.2. X-ray Fluorescence Spectroscopy (XRF)

The chemical composition of the samples was determined through X-ray fluorescence (XRF), using a Rigaku ZSX Primus II XRF spectrometer (WD-XRF ZSX Primus II Rigaku, Tokyo, Japan) with an Rh anode (4.0 kW). Based on the scan results, a semiquantitative analysis was performed using SQX calculation software (version 7.42) and the fundamental parameter method. Prior to the XRF analyses, the loss on ignition value (LOI) was determined by heating the samples to 950 °C and calculating the weight loss. The amount of each element was normalized up to 100% considering the LOI value. To measure the content of carbon (C), an automatic analyzer (EA 1108 Carlo Erba Instruments, Strada Rivoltana, Rodano, Milan, Italy) was used.

#### 2.2.3. Fourier-Transform Infrared Spectroscopy (FTIR)

Infrared spectra were collected using the Nicolet 6700 spectrometer (Thermo Fisher Scientific, Waltham, MA, USA). Accurately, 2 mg of each material was mixed with 200 mg of KBr and pressed to form a pellet. Spectra were collected in the range of 400–4000 cm^−1^ at a 1 cm^−1^ resolution with 64 scan averages.

#### 2.2.4. N_2_ Adsorption–Desorption Studies at 77 K

The specific surface area (S_BET_) and the porosity of the samples were analyzed using the N_2_ gas adsorption–desorption isotherms at 77 K with an ASAP 2020 apparatus (Micromeritics, Norcross, GA, USA). The samples were degassed for 12 h at 378 K. The S_BET_ was calculated using the BET equation [36]. The total pore volume (V_tot_^0.99^) was estimated from the amount of N_2_ adsorbed at a relative vapor pressure (p/p_0_) of ~0.99. The volume of micropores (V_mic_^DR^) was calculated using the Dubinin–Radushkevich method [37]. The mesopore volume (V_mes_^BJH^) was determined from the adsorption branch of the isotherms using the Barrett–Joyner–Halenda (BJH) method [38] in the mesopore range proposed by Dubinin [37]. The macropore volume (V_mac_) was calculated using Equation (1):

V_mac_ = V_tot_^0.99^ − (V_mic_^DR^ + V_mes_^BJH^) (1)

#### 2.2.5. pH at Point of Zero Charge (pHpzc)

The pHpzc value of examined materials was measured according to the method described by Kragović et al. [39]. A 0.01 M sodium nitrate (Avantor, Gliwice, Poland) solution was used as a background electrolyte. The initial pH (pH_i_) of the background electrolyte solution was adjusted to a desired value in the range from 5.5 to 10.5 using 0.1 M NaOH and 0.1 M HNO_3_ solutions (Avantor) and measured using an ELMETRON CX-502 pH meter equipped with a glass electrode (Elmetron, Zabrze, Poland). Accurately, 50 mg of zeolite was weighed into a 50 mL plastic flask and mixed using a laboratory shaker for 24 h with 25 mL of electrolyte solutions of various initial pH. Prior to pH measurement, the samples were centrifuged at 4500 rpm for 5 min. The pHpzc was determined by plotting the difference between the final and the initial pH (ΔpH) against pH_i_. The experiments were performed in duplicate. The presented results are a mean value with a standard deviation of no greater than 1%.

#### 2.2.6. Scanning Electron Microscopy

Scanning electron microscope (SEM) imaging was employed to obtain a better understanding of the textural properties of the investigated materials. Images were made using SEM (JEOL JSM-820, Tokyo, Japan), and the accelerating voltage was 20 kV. To obtain better-quality images, all samples were covered with a gold layer using a JEOL JEE-4X vacuum evaporator.

#### 2.2.7. Leaching Test

The leaching test was conducted at a liquid-to-solid ratio of 1:10 (material:distilled water) in 100 mL Erlenmeyer flasks. Samples were mixed using an orbital shaker (GFL 3015, SciQuip, Wem, UK) for 24 h. Next, the samples were filtered using 0.22 µm membrane filters. The elemental contents were determined according to an ISO standard [40], using an ICP-OES spectrometer (ICP-OES Optima 7300DV, Perkin Elmer, Waltham, MA, USA). The experiments were carried out in triplicate.

#### 2.2.8. Polycyclic Aromatic Hydrocarbons (PAHs) Content

Air-dried NaX–FA, NaX–Ver, and NaX–C samples (7.00 ± 0.02 g) were mixed with 100 mL of n-hexane/acetone, 1:1 (*v*/*v*), (Chempur reagent grade 99%) at room temperature and extracted using FOSS Soxtec Avanti 2055. To all of the samples, 100 μL of deuterated phenanthrene-d10 (Sigma-Aldrich, St. Louis, MO, USA) at a concentration of 2000 µg mL^−1^ in dichloromethane (DCM) as an internal standard was added. The solutions after Soxtec extraction were concentrated to a volume of 0.5 cm^3^ under nitrogen evaporation. The extracts were passed through a column containing activated silica gel topped with 5 g of anhydrous sodium sulfate. The air-dried samples after Soxtec extraction were solved with 4 mL of hexane and eluted with two portions (8 mL) of a mixture of hexane/DCM, 1:1 *v*:*v*, and analyzed by GC-MS (Agilent 7890A GC with 5975C MSD, Santa Clara, CA, USA). The injection was carried out in on-column mode. The transfer line was 300 °C. The temperature program was 99 °C for the initial temperature and 2 °C min^−1^ to 310 °C for 34 min. Gas flow (He) was 1.1 mL min ^−1^. The analysis was conducted with a 60 m column (DB-5MS, 250 µm, and 0.25 µm), using QTM 16-PAHs mix (CRM47930, Sigma-Aldrich) as a standard. The experiments were carried out in triplicate.

#### 2.2.9. Uranium Radionuclide Content

The uranium radionuclide content of the investigated materials was determined according to the method described elsewhere [41], using an alpha spectrometer (Canberra 7401) with a silicon semiconductor passivated implanted planar silicon detector (PIPS). A pressure of 5 Pa was maintained inside the analytical chamber of the alpha spectrometer. Prior to the measurement, 1 g of each zeolite was mineralized in platinum containers using a mixture of acids containing 5 mL HNO_3_ (Avantor), 5 mL HCl (Avantor), 3 mL HF (Avantor) and 1 mL HClO_4_ (Avantor).

### 2.3. Adsorption of Ammonium Ions from Aqueous Solutions

Single-point adsorption experiments were performed to select the suitable conditions of zeolite dose and initial solution pH for the removal of NH_4_^+^ ions from aqueous solutions. Ammonium nitrate (Chempur) of analytical grade was dissolved in distilled water to obtain a concentration of 100 mg (NH_4_^+^) L^−1^. To define the effect of zeolite dose, 50 mL of the solution with a pH adjusted to 7.00 ± 0.05 by adding 0.1 M NaOH or 0.1 M HNO_3_ (Avantor) was stirred with zeolite on a laboratory shaker (GFL 3015, SciQuip, UK) in a 100 mL Erlenmeyer flask. The selected doses of zeolites were 1, 2, 5, or 10 g L^−1^. After 24 h, the solutions were separated from their suspensions using membrane filters (0.22 μm) for the determination of NH_4_^+^ ion concentration. To find the optimal pH of the solution, the procedure was conducted as described above for a zeolite dose of 2 g L^−1^. The pH of the initial solutions was adjusted to 5.00 ± 0.05, 6.00 ± 0.05, 7.00 ± 0.05, or 8.00 ± 0.05. After 24 h of vigorous stirring, the solutions were separated from their suspensions for the determination of NH_4_^+^ ion concentration. The adsorption kinetics were performed in a 250 mL beaker placed on a magnetic stirrer. Briefly, 200 mL of ammonium nitrate solution (100 mg (NH_4_^+^) L^−1^, pH 7.00 ± 0.05) was mixed with 400 mg of zeolite and the samples were collected from the suspension at predefined time intervals and separated using membrane filters (0.22 μm). Throughout the entire duration of the experiment, the samples were collected from the same beaker. For all collected samples the concentration of NH_4_^+^ ions was determined using the standard Nesslerization method and a spectrophotometer (HITACHI U-5100, Hitachi High-Tech Science Corporation, Tokyo, Japan) at a wavelength of 470 nm [42]. All experiments were performed in duplicate and the obtained results were presented as the mean value with a standard deviation of less than 4%.

The adsorbed amount at a specified time was calculated using Equation (2):

(2) $$q_{t}=\frac{C_{0}-C_{t}}{m}*V$$

where *q_t_* (mg g^−1^) is the adsorption capacity at time *t* (min); *C*_0_ and *C_t_* (mg L^−1^) are the initial concentration and at a time (*t*), respectively; *V* (L) is the volume of the solution; and *m* (g) is the mass of adsorbent.

To obtain a better understanding of the adsorption kinetics, three kinetic models were applied for correlation with the experimental data. The pseudo-first-order model [43] in linear form was calculated using Equation (3):

(3) $$\ln\left(q_{exp}-q_{t}\right)=\ln\left(q_{eq}\right)-k_{1}t$$

where *q_exp_*, *q_t_*, and *q_eq_* (mg g^−1^) are the adsorption capacities, as obtained from the *t/q_t_* vs. *t* graph at time *t* (min) and from the equation, respectively; *k*_1_ (min^−1^) is the model constant.

The pseudo-second-order model [44] in linear form was calculated using Equation (4):

(4) $$\frac{t}{q_{t}}=\frac{1}{k_{2}*q_{eq}^{2}}+\frac{t}{q_{eq}}$$

where *q_t_* and *q_eq_* (mg g^−1^) are the adsorption capacities at time *t* (min) and equilibrium, respectively, and *k*_2_ (g mg^−1^ min^−1^) is the model constant.

The adsorption statics experiments were performed in 100 mL Erlenmeyer flasks. Briefly, 50 mL of NH_4_^+^ ion solutions with concentrations varying from 10 to 200 mg (NH_4_^+^) L^−1^ and a pH adjusted to 7.00 ± 0.05 were mixed with 100 mg of zeolite. The solution samples were collected from the suspension using membrane filters (0.22 μm) after 30 min of vigorous stirring. The adsorption capacity at equilibrium was calculated using Equation (5):

(5) $$q_{eq}=\frac{C_{0}-C_{eq}}{m}*V$$

where *q_eq_* (mg g^−1^) is the adsorption capacity at equilibrium, *C*_0_ and *C_eq_* (mg L^−1^) are the initial concentration and concentration at equilibrium, respectively, *V* (L) is the volume of the solution, and *m* (g) is the mass of adsorbent.

The obtained results were correlated with the Langmuir and Freundlich isotherm models. The Langmuir model, which assumes the occurrence of monolayer adsorption over a homogenous surface [45], was calculated using Equation (6):

(6) $$\frac{C_{eq}}{q_{eq}}=\frac{C_{eq}}{q_{max}}+\frac{1}{K_{L}*q_{max}}$$

where *q_eq_* and *q_max_* (mg g^−1^) are the adsorption capacities at equilibrium and maximum adsorption capacity, respectively, *C_eq_* (mg L^−1^) is the equilibrium concentration, and *K_L_* (L mg^−1^) is the model constant attributed to the affinity of active sites for adsorbate.

The Freundlich sorption isotherm assumes the multilayer adsorption on the heterogeneous adsorbent surface [46], and is represented in its linear form by Equation (7):

(7) $$\ln\left(q_{eq}\right)=\ln\left(K_{F}\right)+\frac{1}{n}\ln\left(C_{eq}\right)$$

where *q_eq_* (mg g^−1^) is the adsorption capacity at equilibrium, 1/*n* (-) is the heterogeneity factor, and *K_F_* (mg^1−1/n^ L^1/n^ g^−1^) is the model constant.

### 2.4. Regeneration of Zeolites after Adsorption

To investigate the practical aspect of the application of investigated materials, we analyzed the regeneration of the zeolites after adsorption. Briefly, after adsorption of ammonium ions (zeolite dose: 2 g L^−1^, initial solution concentration: 100 mg (NH_4_^+^) L^−1^, initial pH = 7.00 ± 0.05, time: 30 min), the zeolites were filtered and dried in an oven at 120 °C for 24 h. Next, 50 mg of zeolite was weighed in a 100 mL Erlenmeyer flask and regenerated using 25 mL of 1 M NaCl solution (Avantor) with a pH adjusted to 10.00 ± 0.05 using 1 M NaOH solution (Avantor) for 30 min. After regeneration, the zeolites were washed with distilled water and dried in an oven at 120 °C for 24 h. The procedure of adsorption–desorption was repeated three times to investigate the adsorption efficiency after three cycles. All experiments were performed in duplicate and the obtained results are presented as the mean value with a standard deviation of less than 4%.

## 3. Results

### 3.1. Physical and Chemical Properties of the Materials

#### 3.1.1. X-ray Diffraction (XRD)

**Figure 1:**
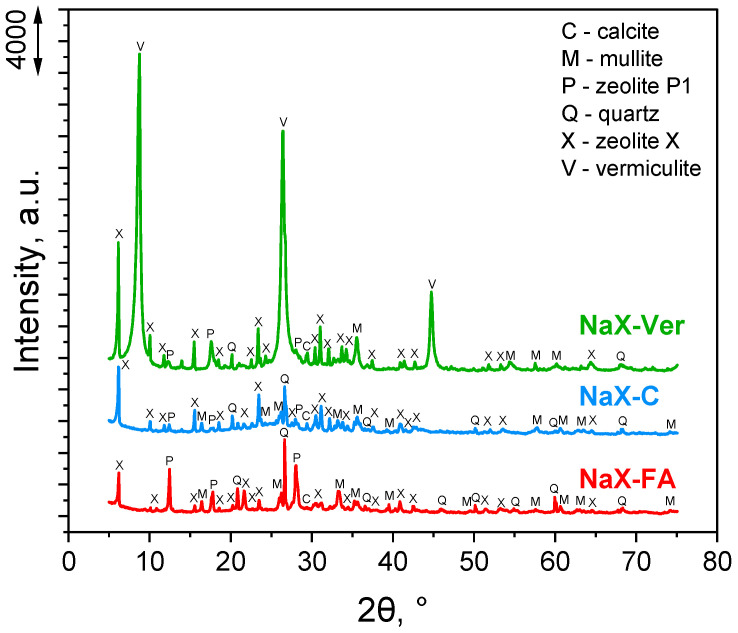
XRD patterns of investigated materials.

The X-ray diffraction patterns of the synthetic zeolites are presented in Figure 1. Na-X phase was recognized by the main d-spacing d_hkl_ = 14.35; 3.79; 5.70; 4.79; 4.38; 7.49; and 2.87 Å. The presence of the zeolite NaP1 phase was confirmed by d_hkl_ = 7.12; 5.00; and 3.18 Å. Zeolite X is the main component in the NaX–FA sample and the zeolite–carbon composite (NaX–C). In the case of the above samples, a small amount of NaP1 zeolite also occurs. Except for the main zeolitic phase, mullite, quartz, and trace amounts of calcite were detected. The main difference between the NaX–FA and NaX–C is a carbonaceous substance, visible in the form of a background, increasing from 15° to 30° 2θ. In the vermiculite–zeolite composite (NaX–Ver), the main component was expanded vermiculite, which was recognized by the strongest reflections d_hkl_ = 10.19; 3.38; and 2.02 Å.

#### 3.1.2. X-ray Fluorescence Spectroscopy (XRF)

The chemical compositions of the investigated samples, as determined by XRF, are listed in Table 1. The main constituents of the zeolites are Si and Al. In the zeolite–carbon composite, carbon has a significant share, represented by the LOI value. What draws attention to the zeolite–vermiculite composite is the content of Mg (14.29 wt.%), which is one of the main elements found in vermiculite.

#### 3.1.3. Fourier-Transform Infrared Spectroscopy (FTIR)

**Figure 2:**
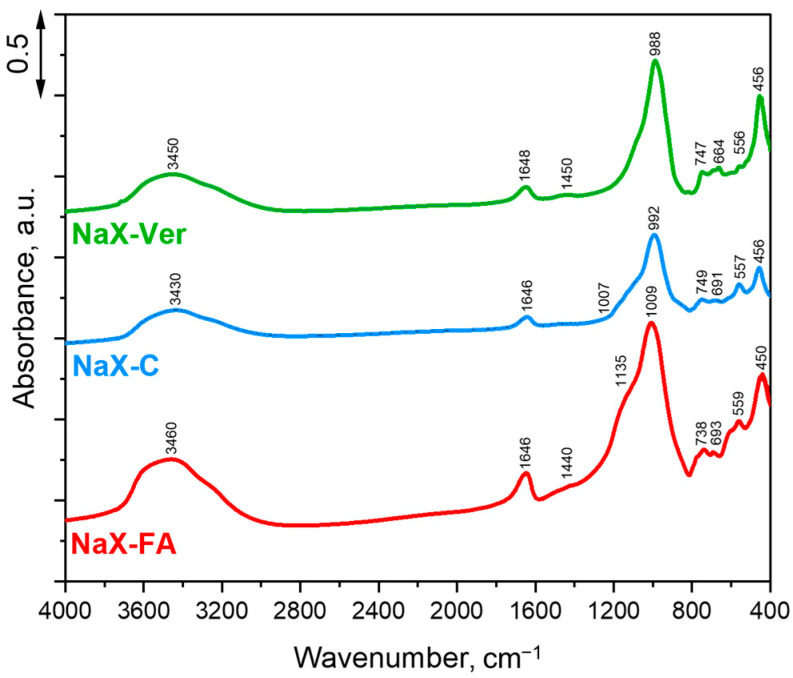
FTIR spectra of investigated zeolites.

The FTIR spectra are presented in Figure 2. The bands at about 3800–3000^−1^ and 1645 cm^−1^ were assigned to stretching and bending vibrations of H_2_O molecules, respectively. Bridging OH^−^ groups were indicated by lower wavenumbers in the case of zeolite NaX–FA and composites NaX–C, NaX–Ver in the range of 3400–3500 cm^−1^, and is a common observation for faujasite-type zeolites [47,48]. All samples contain an admixture of calcite (Figure 1) as the bands attributed to CO_3_^2−^ vibrations are visible at ∼1450 cm^−1^. The peak at 1135 cm^−1^ registered for NaX–FA, NaX–C, and NaX–Ver can originate from the stretching vibration of C-OH [49], the effect of the presence of unburned coal in the composition of the samples. The main bands in the FTIR spectra of the samples were associated with the structural Si–O (Si) and Si–O (Al) tetrahedra vibrations in the range from 1200 to 400 cm^−1^. Bands in the region from 1250 to 920 cm^−1^ were assigned to the internal vibration of the TO_4_ (T = Si or Al) tetrahedra. A high peak at around 990 cm^−1^ refers to a well-defined skeleton of aluminosilicate formed in the samples—the band can be assigned to the asymmetric stretching vibrations of bridge bonds Si–O(Si) and Si–O(Al) in the zeolite and vermiculite frameworks. In the range of 800–500 cm^−1^, so-called pseudo-lattice vibrations originating from over-tetrahedral structural units ([SiO_4_] and [AlO_4_] tetrahedra) can be observed [50]. The bands at about 450 cm^−1^ were assigned to the internal vibrations due to the bending of the T–O tetrahedra [51]. In the case of NaX–Ver, the peak at 456 cm^−1^ is higher compared to the NaX–FA and NaX–C because it is additionally attributed to the bending of Si–O–M vibrations (where M = Al, Fe, Mg, or Si), typically for exfoliated vermiculite [52].

#### 3.1.4. Textural Parameters and pHpzc

**Figure 3:**
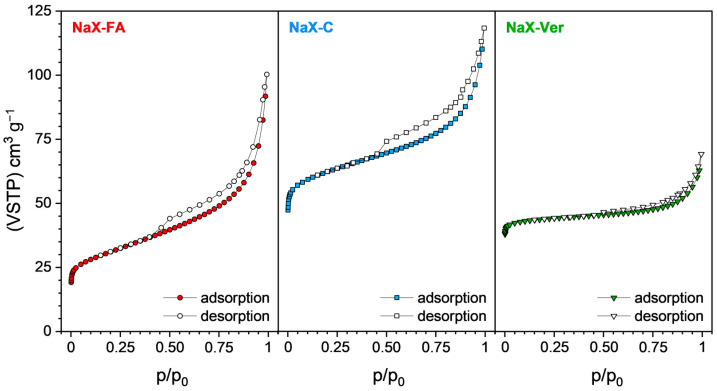
Adsorption–desorption isotherms of N_2_ at 77 K for investigated zeolites.

Textural parameters were determined for NaX–FA zeolite, and NaX–C and NaX–Ver composites (Table 2). All investigated materials NaX–FA, NaX–C, and NaX–Ver have a type IV isotherm with a hysteresis loop of type H3 (Figure 3). This can be related to a mesoporous character and the formation of slit-shaped pores. A significant increase in the nitrogen adsorption at low p/p_0_ observed for the NaX–C indicates a high contribution of micropores. This has been confirmed by the V_mic_ value (Table 2); micropores are 51% of the V_tot_, which reflects the high contribution of fine pores in NaX–C. A much lower contribution of micropores (28%) is attributed to the NaX–FA, where mesopores dominate (65%). The results presented in Table 2 show that the proportion of micropores in this sample is 66%. This indicates the microporous nature of the vermiculite that is part of this composite. The pHpzc values of the zeolites are summarized in Table 2. The pHpzc values were found to be high, probably due to the high amount of mobile alkaline metals (sodium and potassium) as well as alkaline earth metals (calcium and magnesium). The correlation of ΔpH vs. initial pH (pH_i_) is presented in Figure S1.

#### 3.1.5. Scanning Electron Microscopy (SEM)

**Figure 4:**
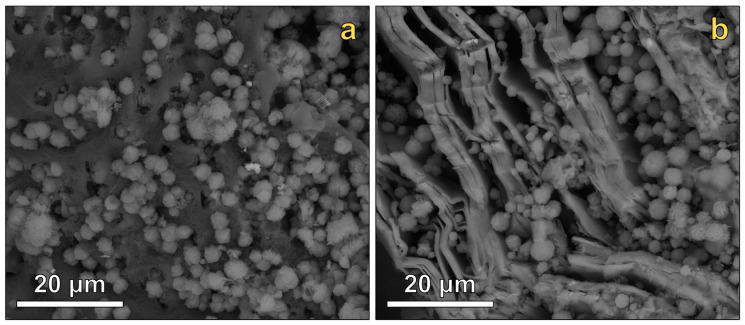
SEM images of composite zeolites: NaX–C (**a**), and NaX–Ver (**b**).

In Figure 4, the SEM images of the zeolites NaX–C and NaX–Ver are presented. As can be seen, the zeolite crystals are well dispersed over the composite surfaces of both NaX–C and NaX–Ver materials. The vast majority of zeolite crystals were characterized by uniform sizes that varied from about 2 to 5 µm. Zeolite is exhibited over the surface as a single crystal instead of aggregates dispersed over a carbon matrix (zeolite NaX–C, Figure 4a) or vermiculite (zeolite NaX–Ver, Figure 4b) [28]. The characteristics of the expanded vermiculite-separated leaf-like structures can be clearly identified in Figure 4b [53].

#### 3.1.6. Safety Assessment (Leaching Test, Contents of PAHs, and Uranium Radionuclides)

The leaching test allowed us to identify the fraction that can be transferred into solution during the contact of the materials with water. The results are summarized in Table 3. For all samples, the greatest amount of identified analyte was sodium and potassium, which, in fact, were also responsible for the high pHpzc of the investigated materials (Table 2). Silica and alumina contents were below 200 µg g^−1^, except in NaX–FA for which the Si content in the leachate was 661.42 µg g^−1^. This might be a result of some unreacted fly ash occurrence within this sample. It can clearly be noticed that the NaX–Ver zeolite had a nearly 2-fold higher amount of sulfur when compared to zeolite NaX–C, which could be a result of the different types of fly ash used for materials synthesis. Heavy metals including Cr, Mo, Cu, Pb, Ni, Co, and Cd were present in rather low amounts < 3 µg g^−1^.

The total content of PAHs (ΣPAHs) based on the number of aromatic rings was examined among investigated materials and presented in Table 3. The ΣPAHs followed the order NaX–Ver < NaX–FA < NaX–C. The highest ΣPAHs (36.11 µg g^−1^) was determined for the NaX–C composite and can be explained by the high content of carbonaceous species within the raw material (HCFA). Such a phenomenon can be the result of incomplete coal combustion, also confirmed by the highest share of 5- and 6-ring PAHs [54]. Among all the investigated materials, the most dominant group was the 4-ring PAHs. The lowest content of 5- and 6-rings, which are the most harmful for living organisms (PAHs with higher molecular weights are considered the most responsible for carcinogenic toxicity when compared to low molecular weight PAHs) and to the environment, was observed for composite NaX–Ver [55]. It should be stated that the determined levels of ΣPAHs are nearly 1000-fold lower when compared to their levels in fly ash (data from the literature), hence the risk of applying zeolites obtained from fly ashes—in terms of PAH content, can be considered negligible [56].

The share of ^234^U and ^238^U is summarized in Table 3 and Figure S2. The concentration of ^234^U and ^238^U in the investigated zeolites varied in the range from 5.93 to 7.94 mBq g^−1^. The reported levels are characteristic of fly ashes obtained from coal combustion in a fluidized bed [57]. Their share and concentration strictly depend on the production technology, particle size, and chemical composition of the coal. It can be concluded that ^234^U was transferred from the fly ash to zeolites during their synthesis. It can be clearly seen that the activity ratio ^234^U/^238^U was close to 1 for NaX–FA and NaX–Ver: 1.04 and 1.07, respectively, implying an equilibrium between isotopes. Composite NaX–Ver showed higher contents of both ^234^U and ^238^U, 29 and 25%, respectively, when compared to NaX–FA, suggesting that uranium was partially transferred with the soluble fraction after NaX–FA synthesis. A slightly lower activity ratio of ^234^U/^238^U observed for zeolite NaX–C (0.85) confirms the different nature of the HCFA used for the zeolite synthesis [58].

### 3.2. Adsorption of Ammonium Ions

#### 3.2.1. Effect of Operation Conditions on NH_4_^+^ Ions Removal

**Figure 5:**
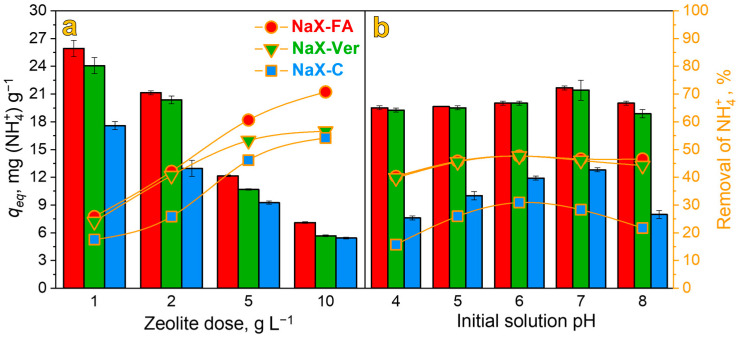
The effect of operating conditions: zeolite dose (**a**) and initial solution pH (**b**) on the adsorption capacity (left Y-axis) and percentage removal (right Y-axis) of ammonium ions. Error bars indicate the standard deviation of duplicate measurements.

The key factors that guide the adsorption process are the adsorbent dose and initial solution pH. In Figure 5a,b, the results of those operating conditions are compared. As can be seen, with an increase in the zeolite dose from 1 to 10 g L^−1^, the adsorption capacity drops with a simultaneous increase in the removal of NH_4_^+^ ions. The efficiency of adsorption was strictly dependent on both zeolite dose and zeolite type. As the zeolite dose increased from 1 to 10 g L^−1^, the removal of NH_4_^+^ ions increased from 25.9, 24.0, and 17.5% to 70.8, 56.6, and 54.2% for zeolites NaX–FA, NaX–Ver, and NaX–C, respectively. This was a result of the increasing number of active sites with the increasing zeolite dose [59]. The most dynamic ramp in the percentage removal of ammonium ions was observed when the dose increased from 1 to 2; however, a further increase in the zeolite dose from 5 to 10 g L^−1^ had a rather weak effect for zeolites NaX–Ver and NaX–C, which were close to saturation. For further analyses, the zeolite dose 2 g L^−1^ was selected as optimal to obtain the balance between both sufficient adsorption capacity of 21.1, 20.4, and 13.0 mg (NH_4_^+^) g^−1^ and percentage removal of 42.2, 40.6, and 25.9 % for NaX–FA, NaX–Ver, and NaX–C, respectively.

As the solution pH increased from 4 to 7, the adsorption capacity increased and reached the maximum at pH 7 for all samples. Next, for a pH of 8, a noticeable drop in adsorption capacity was observed in all zeolite–adsorbate systems. It can be thus assumed that suitable pH conditions for the removal of ammonium ions from aqueous solutions vary from 6 to 7 (maximum % removal of NH_4_^+^ ions at pH 6–47.6, 47.7, and 30.9 % for zeolites NaX–FA, NaX–Ver, and NaX–C, respectively). Such a phenomenon can be also found in the literature, and it implies that the adsorption of ammonium ions is most favorable in the pH range from about 5 to 7 [60]. Lower adsorption capacities at a pH below 6 are explained by the competition of H_3_O^+^ ions with NH_4_^+^ ions in acidic conditions [61]. At a pH below 5, the zeolitic structure can collapse, which implies the importance of this operating parameter in adsorption [60]. On the other hand, at a pH above 8, the ammonia–ammonium ions equilibrium shifts toward the formation of ammonia, which can also lead to a drop in adsorption capacity [61]. Hence, a pH of 7 was selected for further investigations.

#### 3.2.2. Adsorption Kinetic Studies

**Figure 6:**
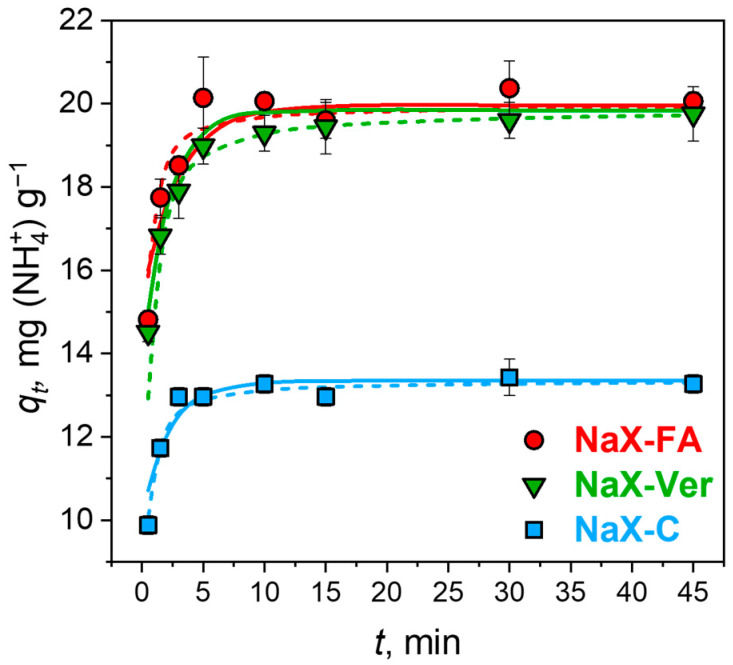
The correlation of experimental data with the adsorption kinetic models: pseudo-first-order (solid line) and pseudo-second-order (dashed line). Error bars indicate the standard deviation of duplicate measurements.

Adsorption kinetic studies allow us to determine the uptake of adsorbate as a function of time, which provides important information for further applications of the adsorbent. The results are summarized in Table 4 and Figure 6. The adsorption of NH_4_^+^ ions was rapid and increased gradually at the very beginning of the process. The adsorption equilibrium value was attained within the first 15 min of the process. Such an observation can be found in the literature and is explained by the high NH_4_^+^ ion concentration gradient in the solution at the initial stage of adsorption [61]. However, the adsorption capacities of materials differ and are strictly dependent on their composition. Overall, the adsorption was governed by the pseudo-second-order kinetic model implying the occurrence of chemisorption. The guiding mechanism in this process is the ion exchange between Na^+^ ions adsorbed on the zeolite surface and the NH_4_^+^ ions in the solution [62]. The mechanism is demonstrated in Equation (8):

Zeolite-Na^+^ + NH_4_^+^_aq._ = Zeolite-NH_4_^+^ + Na^+^_aq._ (8)

Zeolite NaX–FA, in general, poses rather exposed active sites and NH_4_^+^ ions are rapidly able to occupy the adsorption sites [63]. Small NH_4_^+^ ions (~0.331 nm) can be easily transferred through the zeolite pores, improving the adsorption rate [64,65].

#### 3.2.3. Adsorption Statics Studies

**Figure 7:**
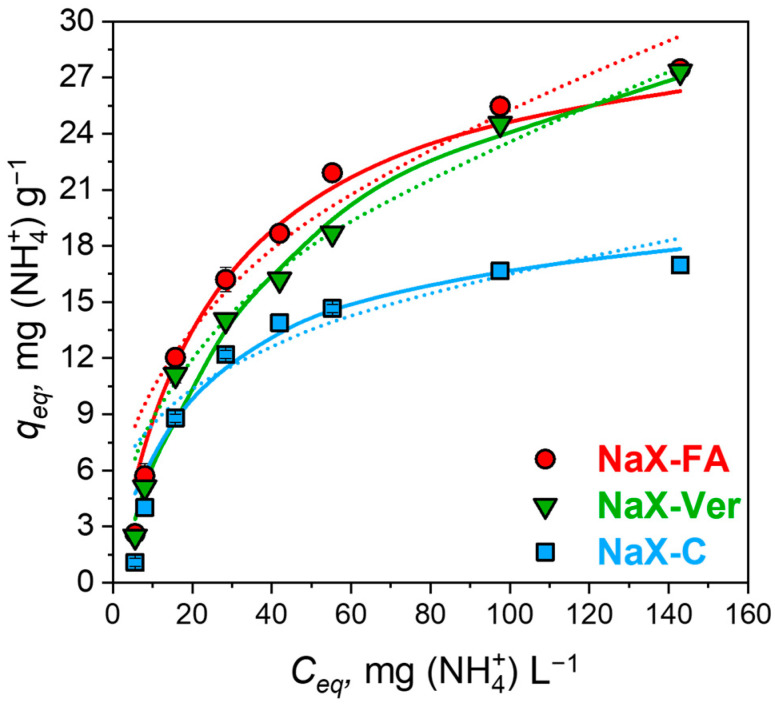
The correlation of adsorption isotherm models: Langmuir (solid line) and Freundlich (dotted line) with experimental data. Error bars indicate the standard deviation of duplicate measurements.

In this study, Langmuir and Freundlich’s static models were employed to fit the adsorption isotherms. The graphical visualization of the results is presented in Figure 7. As the ammonium ion concentration increased, the equilibrium adsorption capacities increased rapidly. It can be also found in the literature [66] that, with increasing concentration of ammonium ions in the solution, the adsorption rate also increases. As can be seen in Table 5, both models correlated with the experimental data; however, the Langmuir model displayed a greater correlation R^2^ > 0.98 for all zeolite–adsorbate systems. Such a phenomenon implies monolayer adsorption on a homogenous surface and that adsorbate has an equal affinity to active sites. The calculated values of *q_max_* increased in order: 21.05 > 38.46 > 40.00 mg g^−1^ for zeolites NaX–C, NaX–Ver, and NaX–FA, respectively. From the Freundlich model, the 1/*n* parameter was calculated and its value varied from 0.314 to 0.507, implying that adsorption was favorable in all systems [63,67]. Zeolites NaX–FA and NaX–Ver exhibited comparable adsorption properties toward ammonium ions, whereas zeolite NaX–C was significantly less efficient. The values of Freundlich (*K_F_*) and Langmuir (*K_L_*) constants appeared not to follow the same order. In the case of zeolite NaX–C, the *K_L_* constant was higher (0.034 L mg^−1^) than for zeolites NaX–FA (0.021 L mg^−1^) and NaX–Ver (0.017 L mg^−1^), whereas the *K_F_* constant decreased in order NaX–FA > NaX–Ver > NaX–C, which, in fact, reflects the adsorption capacity. This phenomenon can be a result of the different energy of adsorption resulting from the type of material and the effect of the presence of carbon within the zeolite NaX–C [68]. The obtained results are compared with the literature data in Table 6.

#### 3.2.4. Regeneration Experiment

**Figure 8:**
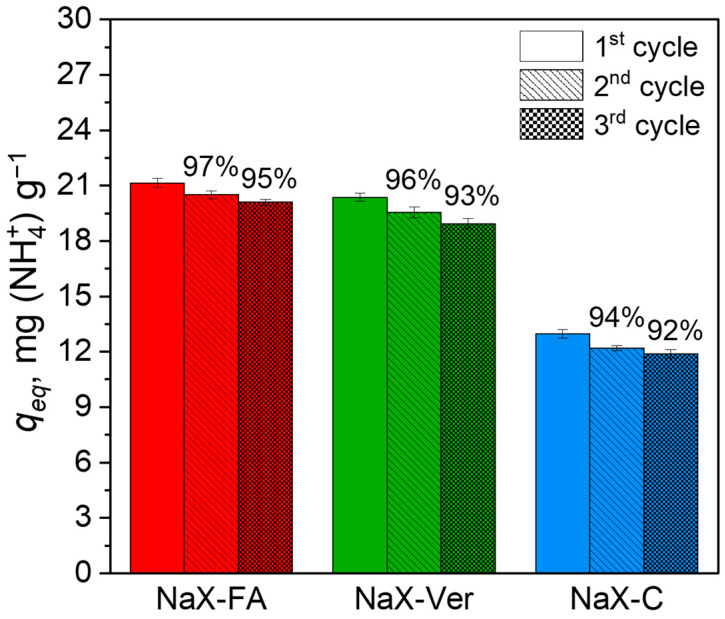
Ammonium ion adsorption capacities after three adsorption cycles. Error bars indicate the standard deviation of duplicate measurements.

The materials were investigated from a perspective of material regeneration and further application as adsorbents of ammonium ions. As can be seen in Figure 8, the efficiency of regeneration was high, and after three cycles, the adsorption capacity toward ammonium ions did not drop dramatically for all investigated zeolites. When compared to initial adsorption capacities, the efficiencies of adsorption of zeolites after three cycles were 95, 93, and 92% for zeolites NaX–FA, NaX–Ver, and NaX–C, respectively. Moreover, the regeneration was rapid and the materials subjected to the cycles did not lose their adsorption properties. Zhang et al. [72] have also proven that the regeneration of zeolites after the adsorption of ammonium ions using NaCl solution was efficient. Hence, the materials can be successfully regenerated and reused after treatment with a NaCl solution of pH 10.00.

### 3.3. Future Perspectives

The evaluation of the chemical composition of zeolites and the composites produced from them is of particular importance regarding their possible future application, especially regarding their potential use in soil amendment. The literature data show that the overall stability of zeolites added to soils is constantly gaining attention [73,74]. To estimate the safety of their application, long-term evaluation tests are required. The potential risk of synthetic zeolite applications mainly involves their long-term stability in the soil system and the long-term stability of heavy metal bonds [22,74,75,76]. The leaching tests conducted in our study revealed generally more components in the NaX–C composite extract, and also in terms of PAH contents (Table 3). Fly ash used to form zeolite is characterized by a very high pHpzc (Table 2). As a result, the zeolite formed from this waste can cause an increase in soil alkalinity. Such behavior can cause a potentially negative effect on both soil and plant ecosystems.

A new indicated direction of research is to investigate the impact of agricultural use of zeolites to reduce greenhouse gas (GHG) emissions. Preliminary research results indicate that the addition of zeolite can mitigate GHG emissions while being in line with the assumptions of sustainable production [77,78]. In the terms of global warming, all actions leading to the reduction in gas emissions are an essential element of the policies for preventing climate change. Furthermore, zeolites have been found to be efficient catalysts in deNOx reactions [79,80,81]; however, limited works are devoted to N_2_O selective catalytic reduction (SCR) [82].

## 4. Conclusions

The presented results have shown a deep insight into both the synthesis and characteristics of zeolite composite materials and the opportunity to utilize harmful and emerging wastes—coal fly ash (CFA) and high carbon coal fly ash (HCFA). The detailed characteristics of obtained materials allowed us to present the chemical composition and textural parameters and, consequently, to estimate the safety of the further application of the materials. For the first time, detailed characteristics of PAH and uranium radionuclide contents were determined in the zeolites obtained from fly ashes. The materials were found to be efficient adsorbents of ammonium ions from aqueous solutions, showing high adsorption capacities of 38.46 and 40.00 mg (NH_4_^+^) g^−1^ for zeolites NaX–Ver and NaX–FA, respectively. Despite the significantly lower adsorption capacity of zeolite NaX–C (21.05 mg (NH_4_^+^) g^−1^), the material shows sufficiently high adsorption properties when compared to the literature data. Most importantly, the materials have shown sufficient regeneration abilities, up to 92–95% of initial adsorption capacity after three cycles. For this reason, zeolite composite materials from CFA and HCFA could become promising tools for the adsorption of various inorganic pollutants.

## Figures and Tables

**Table 1:** Chemical composition of the samples, as obtained from XRF.

Component wt.%	Sample		
	NaX–FA	NaX–C	NaX–Ver
SiO_2_	41.22	22.83	35.68
TiO_2_	1.00	0.78	0.60
Al_2_O_3_	23.87	16.68	19.73
Fe_2_O_3_	7.84	9.64	2.66
CaO	3.33	2.74	2.96
MgO	1.60	1.72	14.29
MnO	0.06	0.11	0.05
BaO	0.08	0.18	0.12
Na_2_O	5.50	3.03	5.21
K_2_O	2.00	1.01	4.19
P_2_O_5_	0.20	0.12	0.03
SO_3_	0.10	0.53	0.16
LOI	12.91	40.36	13.49
Si/Al	1.46	1.16	1.53

LOI—loss on ignition; Si/Al—silica to alumina molar ratio.

**Table 2:** Summary of textural parameters and the pHpzc of investigated zeolites.

Sample	S_BET_ m^2^ g^−1^	V_tot_^0.99^ cm^3^ g^−1^	V_mic_^DR^ cm^3^ g^−1^	V_mes_^BJH^ cm^3^ g^−1^	V_mac_ cm^3^ g^−1^	pHpzc, -
NaX–FA	111.2	0.151	0.043	0.098	0.010	9.85 ± 0.01
NaX–C	237.8	0.177	0.091	0.081	0.005	9.98 ± 0.01
NaX–Ver	178.0	0.102	0.067	0.029	0.009	10.05 ± 0.01

V_tot_^0.99^—total pore volume; V_mic_^DR^—the volume of micropores; V_mes_^BJH^—the volume of mesopores; V_mac_—the volume of macropores; pHpzc—the pH at the point of zero charge.

**Table 3:** Chemical characterization of investigated materials: leaching test, PAH contents, and uranium radionuclides.

Analyte	Sample		
	NaX–FA	NaX–C	NaX–Ver
*Mobile forms (leaching test)*, µg g^−1^ d.m. (ppm)			
Na	5685.35 ± 140.86	3790.32 ± 50.21	4710.35 ± 310.11
K	296.21 ± 10.42	228.00 ± 11.60	114.00 ± 5.87
Al	12.09 ± 3.22	84.00 ± 5.12	25.00 ± 4.53
Si	661.42 ± 36.46	85.50 ± 0.15	171.00 ± 16.30
S	156.67 ± 15.30	79.80 ± 3.10	165.00 ± 18.00
Fe	7.20 ± 1.82	61.50 ± 1.90	18.60 ± 4.05
P	98.98 ± 1.60	57.60 ± 1.28	92.00 ± 9.51
Ca	29.03 ± 3.41	30.40 ± 4.60	23.70 ± 5.05
Mg	5.26 ± 0.86	28.50 ± 1.36	48.60 ± 7.78
Mn	0.09 ± 0.02	1.01 ± 0.01	2.20 ± 0.06
Zn	<0.10	0.99 ± 0.07	0.25 ± 0.10
Cr	2.84 ± 0.08	0.87 ± 0.04	0.54 ± 0.07
Mo	0.84 ± 0.04	0.39 ± 0.02	0.10 ± 0.01
Cu	<0.05	0.33 ± 0.02	0.14 ± 0.02
Pb	<0.05	0.28 ± 0.02	0.07 ± 0.01
Ni	<0.05	0.25 ± 0.02	0.11 ± 0.02
Co	<0.10	0.140 ± 0.004	<0.10
Cd	<0.10	<0.10	<0.10
*Total content of PAHs*, µg g^−1^			
2-rings	0.05 ± 0.00	0.30 ± 0.01	0.01 ± 0.00
3-rings	0.73 ± 0.02	3.15 ± 0.04	0.27 ± 0.01
4-rings	8.11 ± 0.09	29.52 ± 0.27	2.61 ± 0.02
5-rings	0.29 ± 0.01	0.78 ± 0.02	0.08 ± 0.00
6-rings	0.58 ± 0.01	2.36 ± 0.08	0.20 ± 0.01
ΣPAHs	9.77	36.11	3.18
*Content of uranium radionuclides*, mBq g^−1^			
^238^U	5.93 ± 0.49	7.55 ± 0.62	7.44 ± 0.64
^234^U	6.14 ± 0.50	6.39 ± 0.56	7.94 ± 0.66
^234^U/^238^U	1.04	0.85	1.07

±standard deviation, n = 3; d.m.—dry matter.

**Table 4:** Adsorption kinetics parameters of NH_4_^+^ ion adsorption onto zeolites NaX–FA, NaX–Ver, and NaX–C.

Adsorption Kinetics Parameters	Sample		
	NaX–FA	NaX–Ver	NaX–C
*Pseudo-first-order model*			
*q_exp_*, mg g^−1^	19.96	19.84	13.35
*q_eq_*, mg g^−1^	4.69	6.15	3.40
*k*_1_, min^−1^	0.338	0.476	0.506
R^2^	0.882	0.990	0.823
*Pseudo-second-order model*			
*q_eq_*, mg g^−1^	19.96	19.84	13.35
*k*_2_, g mg^−1^ min^−1^	0.386	0.188	0.460
R^2^	0.999	1.000	0.999

T = 25 °C; *t* = 45 min; *C*_0_ = 100 mg (NH_4_^+^) L^−1^; pH = 7 ± 0.05; zeolite dose: 2 g L^−1^.

**Table 5:** Adsorption isotherm parameters of NH_4_^+^ ion adsorption onto zeolites NaX–FA, NaX–Ver, and NaX–C.

Adsorption Isotherm Parameters	Sample		
	NaX–FA	NaX–Ver	NaX–C
*Langmuir isotherm*			
*q_max_*, mg (NH_4_^+^) g^−1^	40.00	38.46	21.05
*K_L_*, L mg^−1^	0.021	0.017	0.034
R^2^	0.99	0.99	0.98
*Freundlich isotherm*			
*K_F_*, mg^1−1/n^ L^1/n^ g^−1^	4.51	3.01	1.592
1/*n*	0.376	0.446	0.507
R^2^	0.97	0.99	0.84

T = 25 °C; *t* = 30 min; *C*_0_ = 10–200 mg (NH_4_^+^) L^−1^; pH = 7.00 ± 0.05; zeolite dose: 2 g L^−1^.

**Table 6:** Comparison of zeolite adsorption capacities toward NH_4_^+^ ions.

Zeolite	Modification of Zeolite	*q_eq_* (Langmuir), mg g^−1^	Adsorption Parameters	Ref.
NaX	Zeolite NaX from fly ash	39.22	Solution concentrations: 20–300 mg (NH_4_^+^) L^−1^; zeolite/solution ratio: 4 g L^−1^.	[63]
NaP1	Zeolite NaP1 from fly ash	25.13	Solution concentrations: 10–250 mg (NH_4_^+^) L^−1^; Zeolite/solution ratio: 2 g L^−1^.	[67]
NZ 3.00 M Na–UW–673 K	NaNO_3_-modified natural zeolite calcinated at 673 K	16.96	Solution concentrations: 1–20 mg (NH_4_^+^) L^−1^; zeolite/solution ratio: 0.3 g L^−1^.	[69]
N-Z	-	33.00	Solution concentrations: 10–5000 mg (NH_4_^+^) L^−1^; zeolite/solution ratio: 10 g L^−1^.	[70]
NZ MOR	Natural zeolite (clinoptilolite) and synthetic zeolite (mordenite)	4.08 2.11	Solution concentrations: 10–100 mg (NH_4_^+^) L^−1^; zeolite/solution ratio: 20 g L^−1^.	[71]
NaY	Zeolite Y in sodium form	26.49	Solution concentrations: 40–615 mg (NH_4_^+^) L^−1^; zeolite/solution ratio: 6.25 g L^−1^.	[33]
NaX–FA NaX–Ver NaX–C	Zeolites from fly ash	40.00 38.46 21.05	Solution concentrations: 10–200 mg (NH_4_^+^) L^−1^; zeolite/solution ratio: 2 g L^−1^.	This study

## Data Availability

Data are available upon reasonable request to authors.

## Supplementary Materials

The following supporting information can be downloaded at: https://www.mdpi.com/article/10.3390/ma16062142/s1, Figure S1: Determination of the pH point of zero charge of investigated zeolites; Figure S2: A spectra of U in the zeolites samples: NaX-FA (a), NaX-C (b), and NaX-Ver (c); Table S1: Chemical composition of CFA, HCFA, and vermiculite as obtained from XRF.
