# Supplementary material for: Zeolite Composite Materials from Fly Ash: An Assessment of Physicochemical and Adsorption Properties
Source: Materials (Basel). 2023 Mar 7;16(6):2142. doi: 10.3390/ma16062142 (PMC10051483; doi:10.3390/ma16062142)
Supplement: Supplementary file 1 [file materials-16-02142-s001.zip › materials-2248431-supplementary.pdf]

SUPPLEMENTARY MATERIALS

Zeolite Composite Materials from Fly Ash: An Assessment of Physicochemical and Adsorption Properties

Jakub Mokrzycki[1*], Wojciech Franus[2], Rafał Panek[2], Maciej Sobczyk[3], Piotr Rusiniak[4], Justyna Szerement[5], Renata Jarosz[3], Lidia Marcińska-Mazur[3], Tomasz Bajda[3], Monika Mierzwa-Hersztek[3,6]

***Corresponding author**: Jakub Mokrzycki, *jmokrzycki@agh.edu.pl*

Table S1. Chemical composition of CFA, HCFA, and vermiculite as obtained from XRF.

Component	Sample		
wt.%	CFA	HCFA	vermiculite
Si	51.6	30.0	42.0
Ti	1.8	1.0	0.9
Al	25.8	13.5	7.8
Fe	7.2	8.6	13.1
Ca	3.0	3.7	1.4
Mg	0.9	1.5	23.0
K	3.0	2.3	0.8
S	0.6	1.2	-
C	-	30.0	-

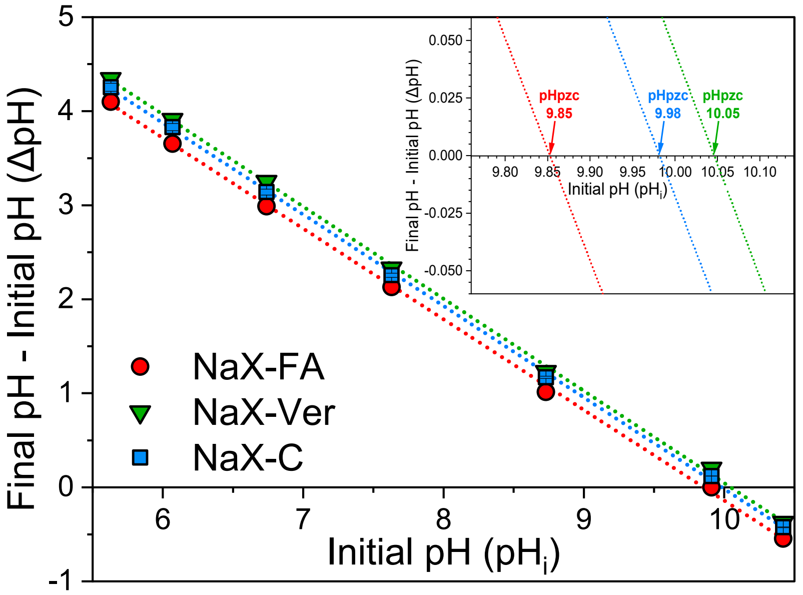

Figure S1. Determination of the pH point of zero charge of investigated zeolites.

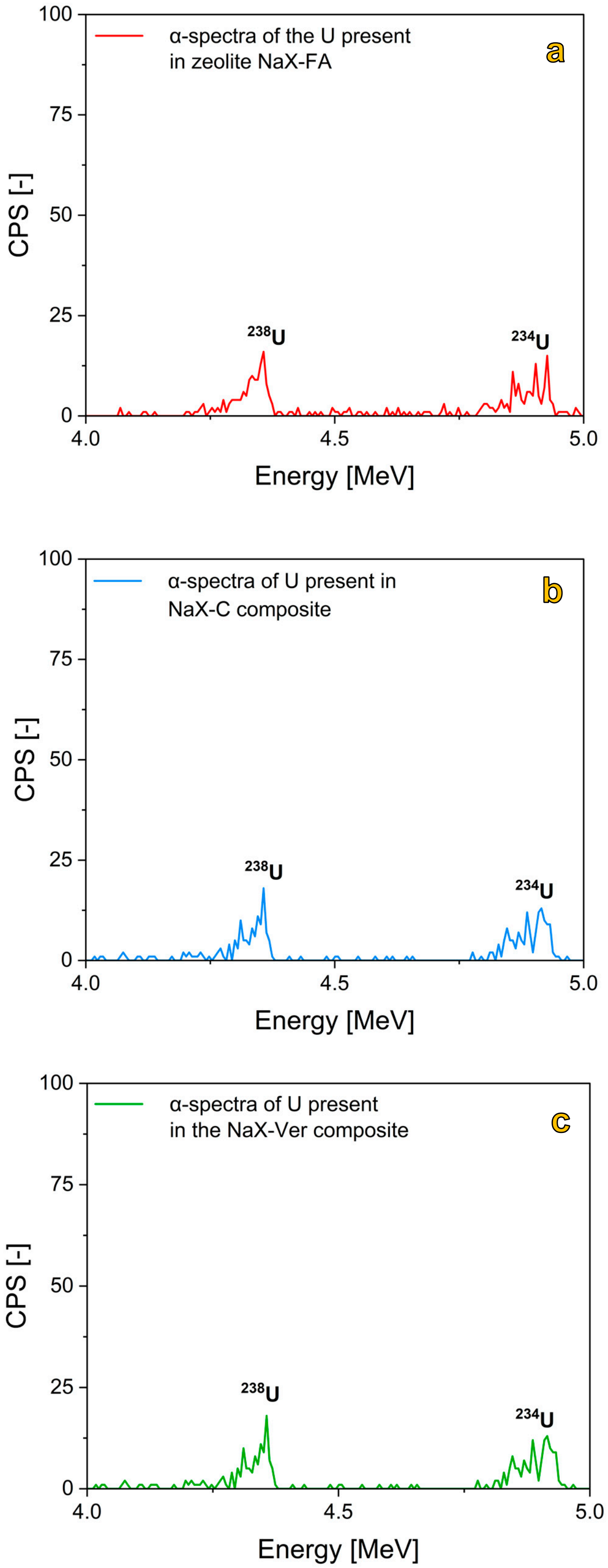

Figure S2. A spectra of U in the zeolites samples: NaX-FA (a), NaX-C (b), and NaX-Ver (c).
